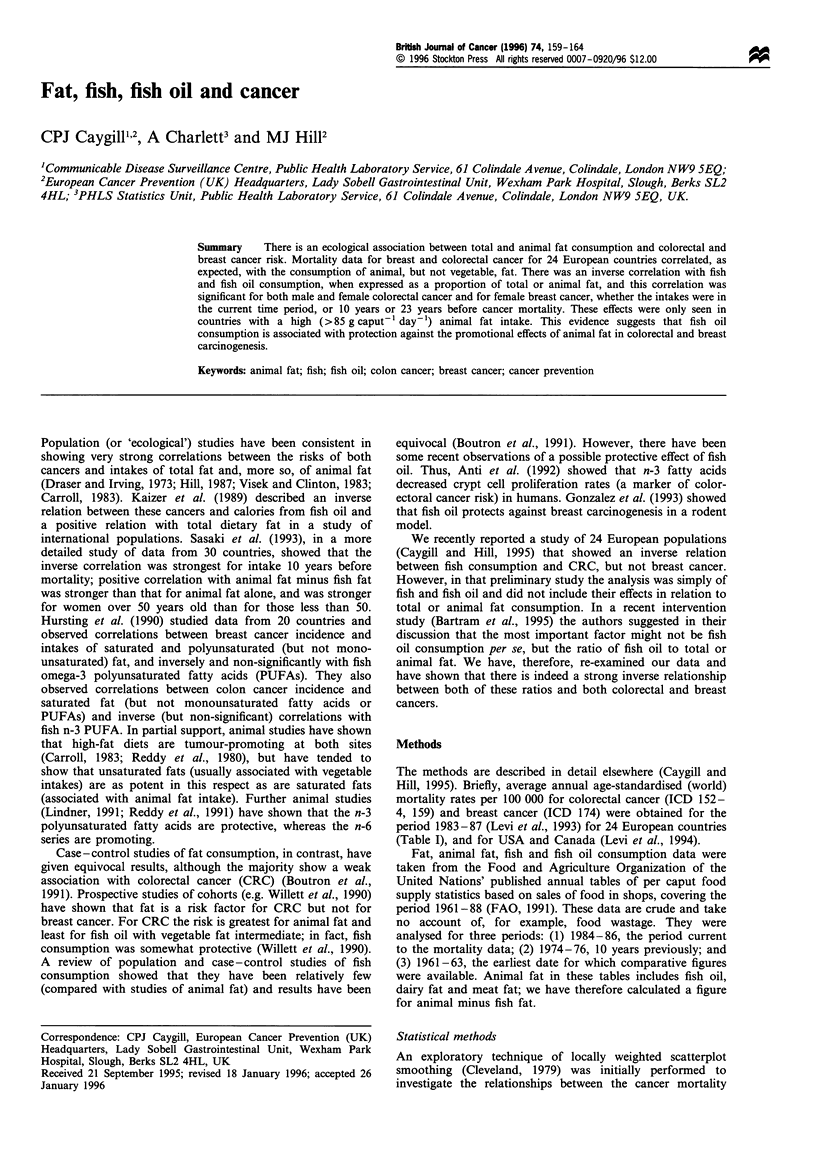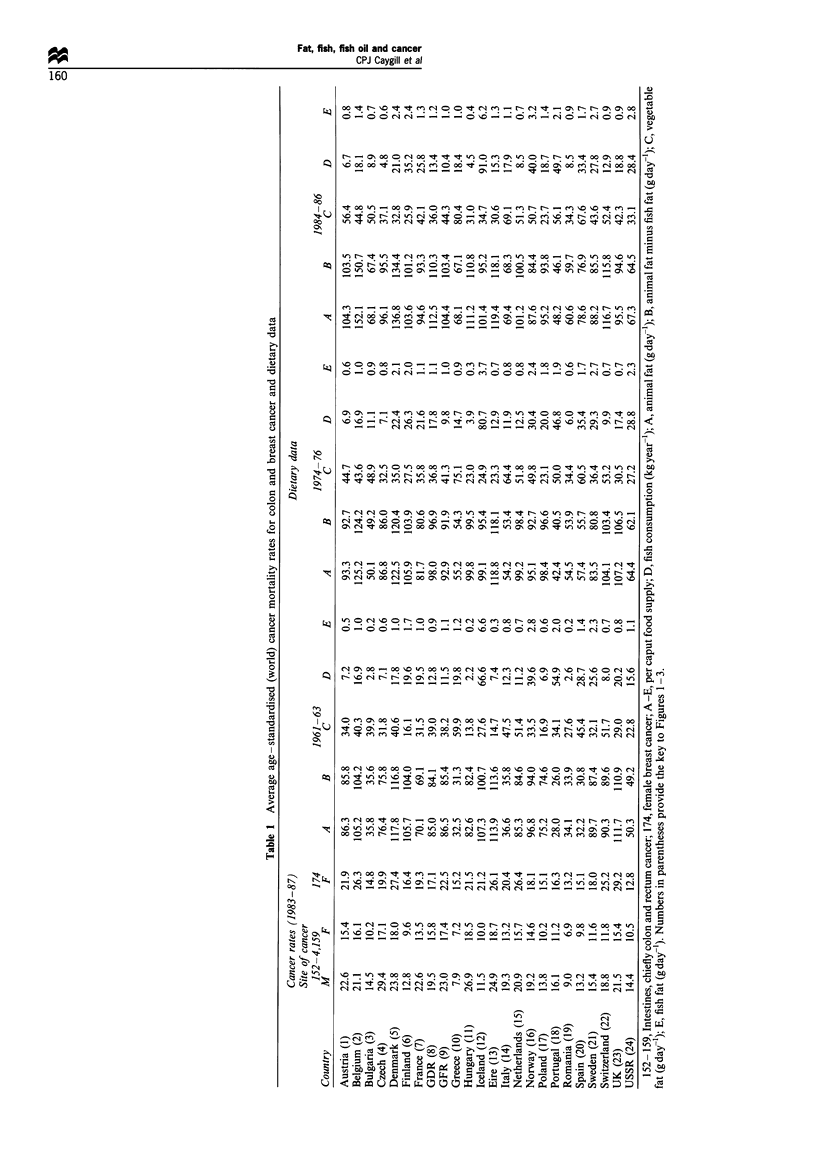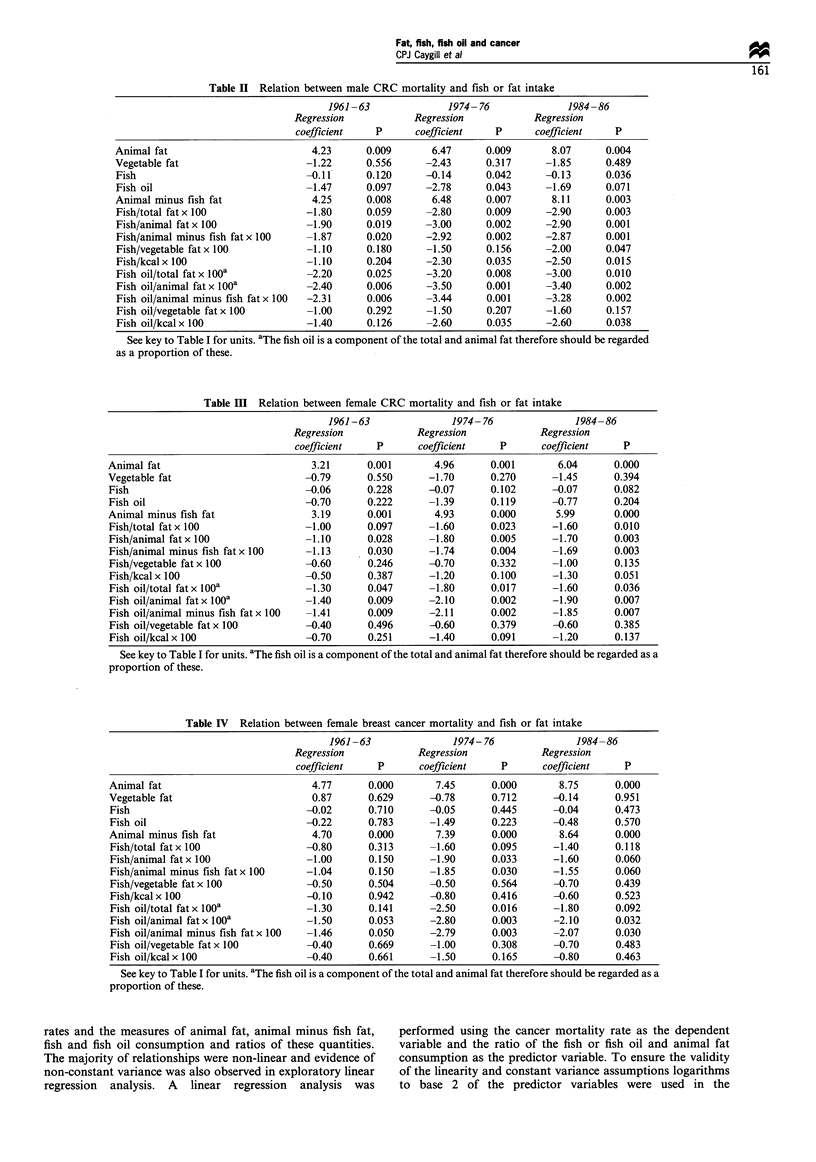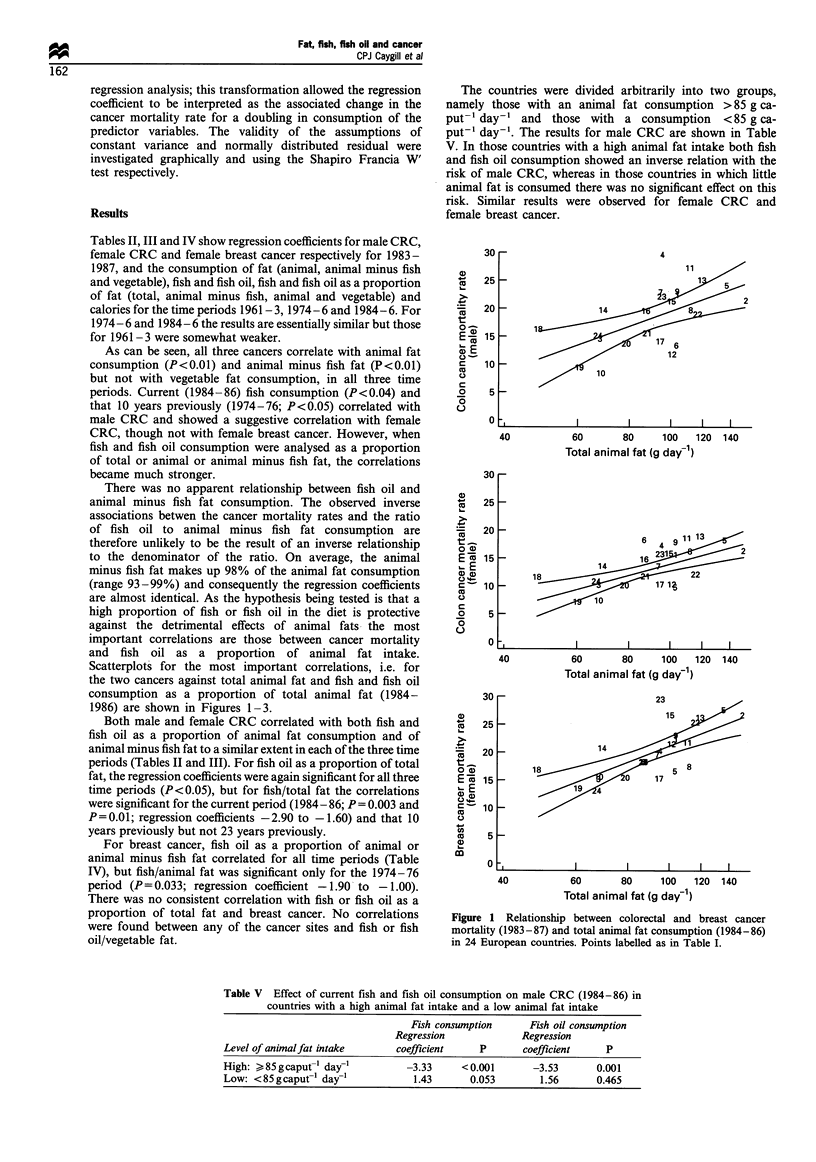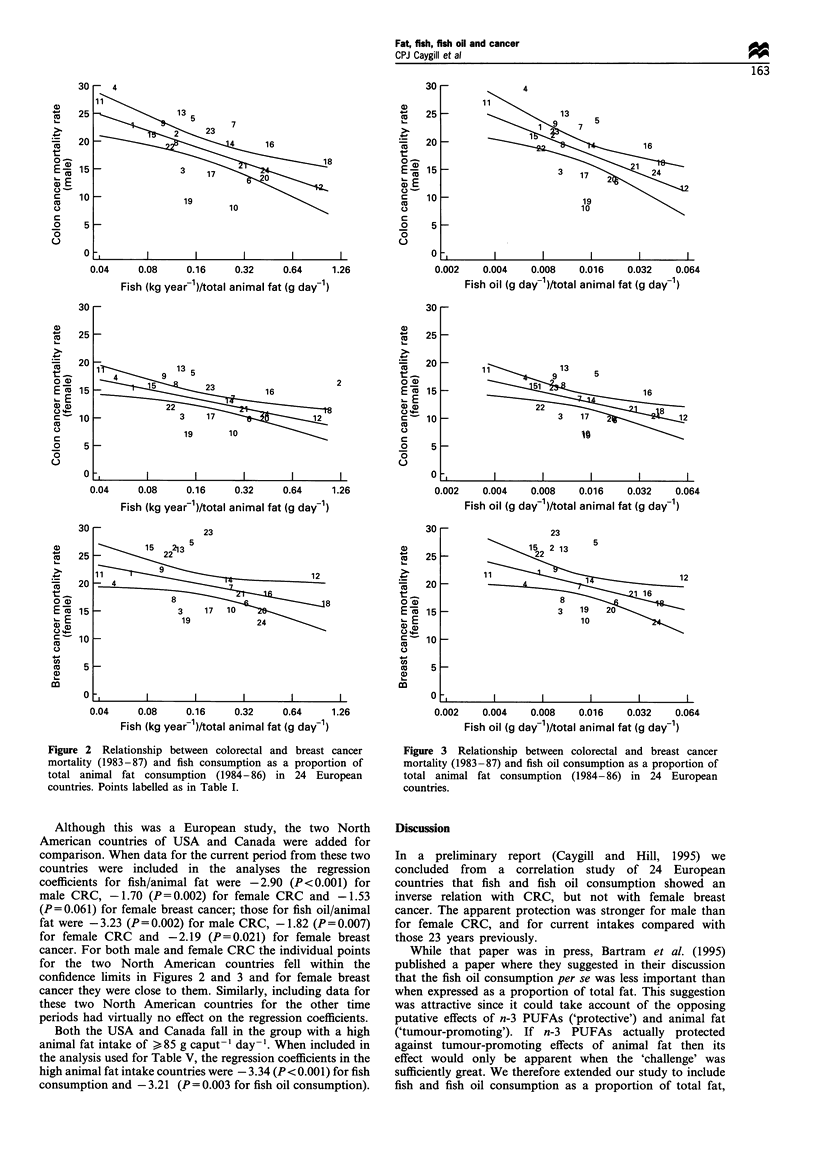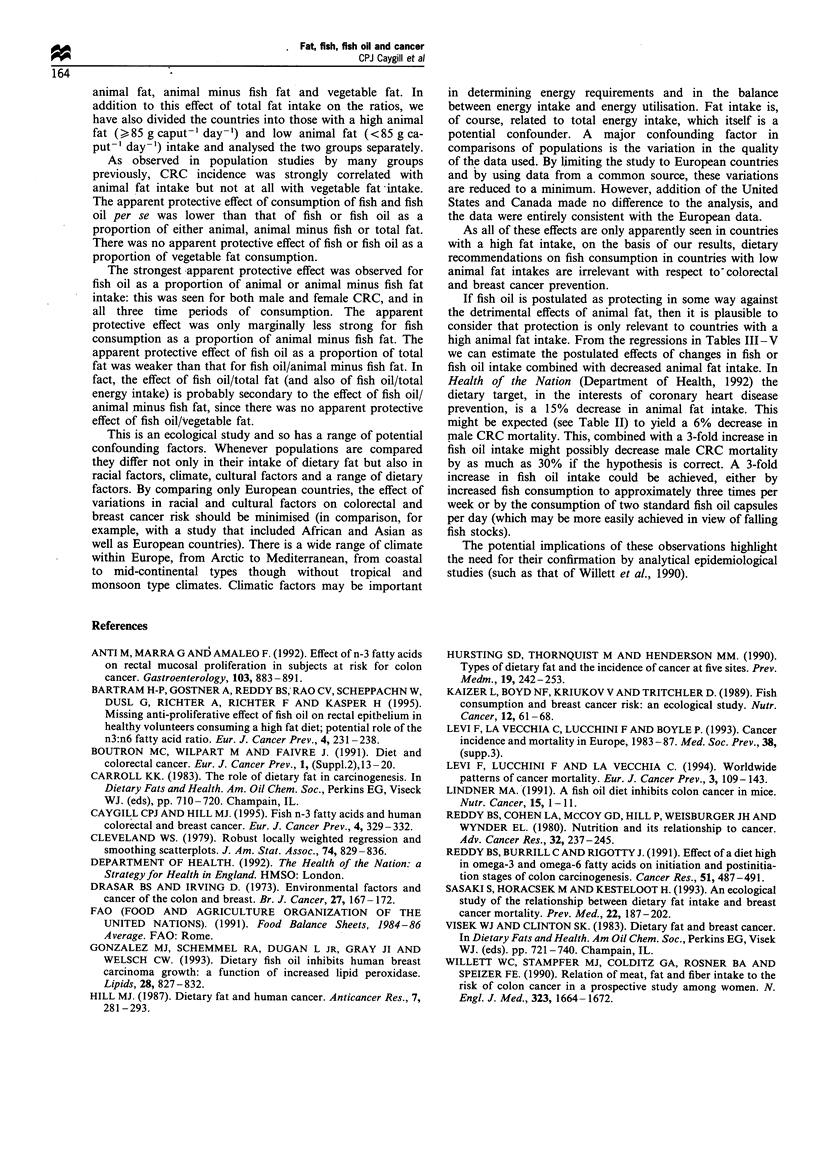# Fat, fish, fish oil and cancer.

**DOI:** 10.1038/bjc.1996.332

**Published:** 1996-07

**Authors:** C. P. Caygill, A. Charlett, M. J. Hill

**Affiliations:** Communicable Disease Surveillance Centre, Public Health Laboratory Service, Colindale, London, UK.

## Abstract

There is an ecological association between total and animal fat consumption and colorectal and breast cancer risk. Mortality data for breast and colorectal cancer for 24 European countries correlated, as expected, with the consumption of animal, but not vegetable, fat. There was an inverse correlation with fish and fish oil consumption, when expressed as a proportion of total or animal fat, and this correlation was significant for both male and female colorectal cancer and for female breast cancer, whether the intakes were in the current time period, or 10 years or 23 years before cancer mortality. These effects were only seen in countries with a high ( > 85 g caput-1 day-1) animal fat intake. This evidence suggests that fish oil consumption is associated with protection against the promotional effects of animal fat in colorectal and breast carcinogenesis.


British Journal of Cancer (1996) 74, 159-164
© 1996 Stockton Press All rights reserved 0007-0920/96 $12.00

### Fat, fish, fish oil and cancer

CPJ Caygill[1,2], A Charlett[3] and MJ Hill[2]

[1]*Communicable Disease Surveillance Centre, Public Health Laboratory Service, 61 Colindale Avenue, Colindale, London NW9 5EQ;* [2]*European Cancer Prevention (UK) Headquarters, Lady Sobell Gastrointestinal Unit, Wexham Park Hospital, Slough, Berks SL2 4HL;* [3]*PHLS Statistics Unit, Public Health Laboratory Service, 61 Colindale Avenue, Colindale, London NW9 5EQ, UK.*

**Summary** There is an ecological association between total and animal fat consumption and colorectal and breast cancer risk. Mortality data for breast and colorectal cancer for 24 European countries correlated, as expected, with the consumption of animal, but not vegetable, fat. There was an inverse correlation with fish and fish oil consumption, when expressed as a proportion of total or animal fat, and this correlation was significant for both male and female colorectal cancer and for female breast cancer, whether the intakes were in the current time period, or 10 years or 23 years before cancer mortality. These effects were only seen in countries with a high (>85 g caput$^{-1}$ day$^{-1}$) animal fat intake. This evidence suggests that fish oil consumption is associated with protection against the promotional effects of animal fat in colorectal and breast carcinogenesis.

**Keywords:** animal fat; fish; fish oil; colon cancer; breast cancer; cancer prevention

Population (or 'ecological') studies have been consistent in showing very strong correlations between the risks of both cancers and intakes of total fat and, more so, of animal fat (Draser and Irving, 1973; Hill, 1987; Visek and Clinton, 1983; Carroll, 1983). Kaizer *et al.* (1989) described an inverse relation between these cancers and calories from fish oil and a positive relation with total dietary fat in a study of international populations. Sasaki *et al.* (1993), in a more detailed study of data from 30 countries, showed that the inverse correlation was strongest for intake 10 years before mortality; positive correlation with animal fat minus fish fat was stronger than that for animal fat alone, and was stronger for women over 50 years old than for those less than 50. Hursting *et al.* (1990) studied data from 20 countries and observed correlations between breast cancer incidence and intakes of saturated and polyunsaturated (but not mono-unsaturated) fat, and inversely and non-significantly with fish omega-3 polyunsaturated fatty acids (PUFAs). They also observed correlations between colon cancer incidence and saturated fat (but not monounsaturated fatty acids or PUFAs) and inverse (but non-significant) correlations with fish n-3 PUFA. In partial support, animal studies have shown that high-fat diets are tumour-promoting at both sites (Carroll, 1983; Reddy *et al.*, 1980), but have tended to show that unsaturated fats (usually associated with vegetable intakes) are as potent in this respect as are saturated fats (associated with animal fat intake). Further animal studies (Lindner, 1991; Reddy *et al.*, 1991) have shown that the *n*-3 polyunsaturated fatty acids are protective, whereas the *n*-6 series are promoting.

Case-control studies of fat consumption, in contrast, have given equivocal results, although the majority show a weak association with colorectal cancer (CRC) (Boutron *et al.*, 1991). Prospective studies of cohorts (e.g. Willett *et al.*, 1990) have shown that fat is a risk factor for CRC but not for breast cancer. For CRC the risk is greatest for animal fat and least for fish oil with vegetable fat intermediate; in fact, fish consumption was somewhat protective (Willett *et al.*, 1990). A review of population and case-control studies of fish consumption showed that they have been relatively few (compared with studies of animal fat) and results have been equivocal (Boutron *et al.*, 1991). However, there have been some recent observations of a possible protective effect of fish oil. Thus, Anti *et al.* (1992) showed that *n*-3 fatty acids decreased crypt cell proliferation rates (a marker of colorectoral cancer risk) in humans. Gonzalez *et al.* (1993) showed that fish oil protects against breast carcinogenesis in a rodent model.

We recently reported a study of 24 European populations (Caygill and Hill, 1995) that showed an inverse relation between fish consumption and CRC, but not breast cancer. However, in that preliminary study the analysis was simply of fish and fish oil and did not include their effects in relation to total or animal fat consumption. In a recent intervention study (Bartram *et al.*, 1995) the authors suggested in their discussion that the most important factor might not be fish oil consumption *per se*, but the ratio of fish oil to total or animal fat. We have, therefore, re-examined our data and have shown that there is indeed a strong inverse relationship between both of these ratios and both colorectal and breast cancers.

### Methods

The methods are described in detail elsewhere (Caygill and Hill, 1995). Briefly, average annual age-standardised (world) mortality rates per 100 000 for colorectal cancer (ICD 152-4, 159) and breast cancer (ICD 174) were obtained for the period 1983-87 (Levi *et al.*, 1993) for 24 European countries (Table I), and for USA and Canada (Levi *et al.*, 1994).

Fat, animal fat, fish and fish oil consumption data were taken from the Food and Agriculture Organization of the United Nations' published annual tables of per caput food supply statistics based on sales of food in shops, covering the period 1961-88 (FAO, 1991). These data are crude and take no account of, for example, food wastage. They were analysed for three periods: (1) 1984-86, the period current to the mortality data; (2) 1974-76, 10 years previously; and (3) 1961-63, the earliest date for which comparative figures were available. Animal fat in these tables includes fish oil, dairy fat and meat fat; we have therefore calculated a figure for animal minus fish fat.

### *Statistical methods*

An exploratory technique of locally weighted scatterplot smoothing (Cleveland, 1979) was initially performed to investigate the relationships between the cancer mortality

Correspondence: CPJ Caygill, European Cancer Prevention (UK) Headquarters, Lady Sobell Gastrointestinal Unit, Wexham Park Hospital, Slough, Berks SL2 4HL, UK
Received 21 September 1995; revised 18 January 1996; accepted 26 January 1996

**Table 1** Average age-standardised (world) cancer mortality rates for colon and breast cancer and dietary data

| Country | Cancer rates (1983–87) Site of cancer 152–4,159 M | F | 174 F | Dietary data 1961–63 A | B | C | D | E | 1974–76 A | B | C | D | E | 1984–86 A | B | C | D | E |
|---|---|---|---|---|---|---|---|---|---|---|---|---|---|---|---|---|---|---|
| Austria (1) | 22.6 | 15.4 | 21.9 | 86.3 | 85.8 | 34.0 | 7.2 | 0.5 | 93.3 | 92.7 | 44.7 | 6.9 | 0.6 | 104.3 | 103.5 | 56.4 | 6.7 | 0.8 |
| Belgium (2) | 21.1 | 16.1 | 26.3 | 105.2 | 104.2 | 40.3 | 16.9 | 1.0 | 125.2 | 124.2 | 43.6 | 16.9 | 1.0 | 152.1 | 150.7 | 44.8 | 18.1 | 1.4 |
| Bulgaria (3) | 14.5 | 10.2 | 14.8 | 35.8 | 35.6 | 39.9 | 2.8 | 0.2 | 50.1 | 49.2 | 48.9 | 11.1 | 0.9 | 68.1 | 67.4 | 50.5 | 8.9 | 0.7 |
| Czech (4) | 29.4 | 17.1 | 19.9 | 76.4 | 75.8 | 31.8 | 7.1 | 0.6 | 86.8 | 86.0 | 32.5 | 7.1 | 0.8 | 96.1 | 95.5 | 37.1 | 4.8 | 0.6 |
| Denmark (5) | 23.8 | 18.0 | 27.4 | 117.8 | 116.8 | 40.6 | 17.8 | 1.0 | 122.5 | 120.4 | 35.0 | 22.4 | 2.1 | 136.8 | 134.4 | 32.8 | 21.0 | 2.4 |
| Finland (6) | 12.8 | 9.6 | 16.4 | 105.7 | 104.0 | 16.1 | 19.6 | 1.7 | 105.9 | 103.9 | 27.5 | 26.3 | 2.0 | 103.6 | 101.2 | 25.9 | 35.2 | 2.4 |
| France (7) | 22.6 | 13.5 | 19.3 | 70.1 | 69.1 | 31.5 | 19.5 | 1.0 | 81.7 | 80.6 | 35.8 | 21.6 | 1.1 | 94.6 | 93.3 | 42.1 | 25.8 | 1.3 |
| GDR (8) | 19.5 | 15.8 | 17.1 | 85.0 | 84.1 | 39.0 | 12.8 | 0.9 | 98.0 | 96.9 | 36.8 | 17.8 | 1.1 | 112.5 | 110.3 | 36.0 | 13.4 | 1.2 |
| GFR (9) | 23.0 | 17.4 | 22.5 | 86.5 | 85.4 | 38.2 | 11.5 | 1.1 | 92.9 | 91.9 | 41.3 | 9.8 | 1.0 | 104.4 | 103.4 | 44.3 | 10.4 | 1.0 |
| Greece (10) | 7.9 | 7.2 | 15.2 | 32.5 | 31.3 | 59.9 | 19.8 | 1.2 | 55.2 | 54.3 | 75.1 | 14.7 | 0.9 | 68.1 | 67.1 | 80.4 | 18.4 | 1.0 |
| Hungary (11) | 26.9 | 18.5 | 21.5 | 82.6 | 82.4 | 13.8 | 2.2 | 0.2 | 99.8 | 99.5 | 23.0 | 3.9 | 0.3 | 111.2 | 110.8 | 31.0 | 4.5 | 0.4 |
| Iceland (12) | 11.5 | 10.0 | 21.2 | 107.3 | 100.7 | 27.6 | 66.6 | 6.6 | 99.1 | 95.4 | 24.9 | 80.7 | 3.7 | 101.4 | 95.2 | 34.7 | 91.0 | 6.2 |
| Eire (13) | 24.9 | 18.7 | 26.1 | 113.9 | 113.6 | 14.7 | 7.4 | 0.3 | 118.8 | 118.1 | 23.3 | 12.9 | 0.7 | 119.4 | 118.1 | 30.6 | 15.3 | 1.3 |
| Italy (14) | 19.3 | 13.2 | 20.4 | 36.6 | 35.8 | 47.5 | 12.3 | 0.8 | 54.2 | 53.4 | 64.4 | 11.9 | 0.8 | 69.4 | 68.3 | 69.1 | 17.9 | 1.1 |
| Netherlands (15) | 20.9 | 15.7 | 26.4 | 85.3 | 84.6 | 51.4 | 11.2 | 0.7 | 99.2 | 98.4 | 51.8 | 12.5 | 0.8 | 101.2 | 100.5 | 51.3 | 8.5 | 0.7 |
| Norway (16) | 19.2 | 14.6 | 18.1 | 96.8 | 94.0 | 33.5 | 39.6 | 2.8 | 95.1 | 92.7 | 49.8 | 30.4 | 2.4 | 87.6 | 84.4 | 50.7 | 40.0 | 3.2 |
| Poland (17) | 13.8 | 10.2 | 15.1 | 75.2 | 74.6 | 16.9 | 6.9 | 0.6 | 98.4 | 96.6 | 23.1 | 20.0 | 1.8 | 95.2 | 93.8 | 23.7 | 18.7 | 1.4 |
| Portugal (18) | 16.1 | 11.2 | 16.3 | 28.0 | 26.0 | 34.1 | 54.9 | 2.0 | 42.4 | 40.5 | 50.0 | 46.8 | 1.9 | 48.2 | 46.1 | 56.1 | 49.7 | 2.1 |
| Romania (19) | 9.0 | 6.9 | 13.2 | 34.1 | 33.9 | 27.6 | 2.6 | 0.2 | 54.5 | 53.9 | 34.4 | 6.0 | 0.6 | 60.6 | 59.7 | 34.3 | 8.5 | 0.9 |
| Spain (20) | 13.2 | 9.8 | 15.1 | 32.2 | 30.8 | 45.4 | 28.7 | 1.4 | 57.4 | 55.7 | 60.5 | 35.4 | 1.7 | 78.6 | 76.9 | 67.6 | 33.4 | 1.7 |
| Sweden (21) | 15.4 | 11.6 | 18.0 | 89.7 | 87.4 | 32.1 | 25.6 | 2.3 | 83.5 | 80.8 | 36.4 | 29.3 | 2.7 | 88.2 | 85.5 | 43.6 | 27.8 | 2.7 |
| Switzerland (22) | 18.8 | 11.8 | 25.2 | 90.3 | 89.6 | 51.7 | 8.0 | 0.7 | 104.1 | 103.4 | 53.2 | 9.9 | 0.7 | 116.7 | 115.8 | 52.4 | 12.9 | 0.9 |
| UK (23) | 21.5 | 15.4 | 29.2 | 111.7 | 110.9 | 29.0 | 20.2 | 0.8 | 107.2 | 106.5 | 30.5 | 17.4 | 0.7 | 95.5 | 94.6 | 42.3 | 18.8 | 0.9 |
| USSR (24) | 14.4 | 10.5 | 12.8 | 50.3 | 49.2 | 22.8 | 15.6 | 1.1 | 64.4 | 62.1 | 27.2 | 28.8 | 2.3 | 67.3 | 64.5 | 33.1 | 28.4 | 2.8 |

152–159, Intestines, chiefly colon and rectum cancer; 174, female breast cancer; A–E, per caput food supply; D, fish consumption (kg year$^{-1}$); A, animal fat (g day$^{-1}$); B, animal fat minus fish fat (g day$^{-1}$); C, vegetable fat (g day$^{-1}$); E, fish fat (g day$^{-1}$). Numbers in parentheses provide the key to Figures 1–3.

**Table II** Relation between male CRC mortality and fish or fat intake

| | *1961–63* Regression coefficient | P | *1974–76* Regression coefficient | P | *1984–86* Regression coefficient | P |
|---|---|---|---|---|---|---|
| Animal fat | 4.23 | 0.009 | 6.47 | 0.009 | 8.07 | 0.004 |
| Vegetable fat | -1.22 | 0.556 | -2.43 | 0.317 | -1.85 | 0.489 |
| Fish | -0.11 | 0.120 | -0.14 | 0.042 | -0.13 | 0.036 |
| Fish oil | -1.47 | 0.097 | -2.78 | 0.043 | -1.69 | 0.071 |
| Animal minus fish fat | 4.25 | 0.008 | 6.48 | 0.007 | 8.11 | 0.003 |
| Fish/total fat x 100 | -1.80 | 0.059 | -2.80 | 0.009 | -2.90 | 0.003 |
| Fish/animal fat x 100 | -1.90 | 0.019 | -3.00 | 0.002 | -2.90 | 0.001 |
| Fish/animal minus fish fat x 100 | -1.87 | 0.020 | -2.92 | 0.002 | -2.87 | 0.001 |
| Fish/vegetable fat x 100 | -1.10 | 0.180 | -1.50 | 0.156 | -2.00 | 0.047 |
| Fish/kcal x 100 | -1.10 | 0.204 | -2.30 | 0.035 | -2.50 | 0.015 |
| Fish oil/total fat x 100[a] | -2.20 | 0.025 | -3.20 | 0.008 | -3.00 | 0.010 |
| Fish oil/animal fat x 100[a] | -2.40 | 0.006 | -3.50 | 0.001 | -3.40 | 0.002 |
| Fish oil/animal minus fish fat x 100 | -2.31 | 0.006 | -3.44 | 0.001 | -3.28 | 0.002 |
| Fish oil/vegetable fat x 100 | -1.00 | 0.292 | -1.50 | 0.207 | -1.60 | 0.157 |
| Fish oil/kcal x 100 | -1.40 | 0.126 | -2.60 | 0.035 | -2.60 | 0.038 |

See key to Table I for units. [a]The fish oil is a component of the total and animal fat therefore should be regarded as a proportion of these.

**Table III** Relation between female CRC mortality and fish or fat intake

| | *1961–63* Regression coefficient | P | *1974–76* Regression coefficient | P | *1984–86* Regression coefficient | P |
|---|---|---|---|---|---|---|
| Animal fat | 3.21 | 0.001 | 4.96 | 0.001 | 6.04 | 0.000 |
| Vegetable fat | -0.79 | 0.550 | -1.70 | 0.270 | -1.45 | 0.394 |
| Fish | -0.06 | 0.228 | -0.07 | 0.102 | -0.07 | 0.082 |
| Fish oil | -0.70 | 0.222 | -1.39 | 0.119 | -0.77 | 0.204 |
| Animal minus fish fat | 3.19 | 0.001 | 4.93 | 0.000 | 5.99 | 0.000 |
| Fish/total fat x 100 | -1.00 | 0.097 | -1.60 | 0.023 | -1.60 | 0.010 |
| Fish/animal fat x 100 | -1.10 | 0.028 | -1.80 | 0.005 | -1.70 | 0.003 |
| Fish/animal minus fish fat x 100 | -1.13 | 0.030 | -1.74 | 0.004 | -1.69 | 0.003 |
| Fish/vegetable fat x 100 | -0.60 | 0.246 | -0.70 | 0.332 | -1.00 | 0.135 |
| Fish/kcal x 100 | -0.50 | 0.387 | -1.20 | 0.100 | -1.30 | 0.051 |
| Fish oil/total fat x 100[a] | -1.30 | 0.047 | -1.80 | 0.017 | -1.60 | 0.036 |
| Fish oil/animal fat x 100[a] | -1.40 | 0.009 | -2.10 | 0.002 | -1.90 | 0.007 |
| Fish oil/animal minus fish fat x 100 | -1.41 | 0.009 | -2.11 | 0.002 | -1.85 | 0.007 |
| Fish oil/vegetable fat x 100 | -0.40 | 0.496 | -0.60 | 0.379 | -0.60 | 0.385 |
| Fish oil/kcal x 100 | -0.70 | 0.251 | -1.40 | 0.091 | -1.20 | 0.137 |

See key to Table I for units. [a]The fish oil is a component of the total and animal fat therefore should be regarded as a proportion of these.

**Table IV** Relation between female breast cancer mortality and fish or fat intake

| | *1961–63* Regression coefficient | P | *1974–76* Regression coefficient | P | *1984–86* Regression coefficient | P |
|---|---|---|---|---|---|---|
| Animal fat | 4.77 | 0.000 | 7.45 | 0.000 | 8.75 | 0.000 |
| Vegetable fat | 0.87 | 0.629 | -0.78 | 0.712 | -0.14 | 0.951 |
| Fish | -0.02 | 0.710 | -0.05 | 0.445 | -0.04 | 0.473 |
| Fish oil | -0.22 | 0.783 | -1.49 | 0.223 | -0.48 | 0.570 |
| Animal minus fish fat | 4.70 | 0.000 | 7.39 | 0.000 | 8.64 | 0.000 |
| Fish/total fat x 100 | -0.80 | 0.313 | -1.60 | 0.095 | -1.40 | 0.118 |
| Fish/animal fat x 100 | -1.00 | 0.150 | -1.90 | 0.033 | -1.60 | 0.060 |
| Fish/animal minus fish fat x 100 | -1.04 | 0.150 | -1.85 | 0.030 | -1.55 | 0.060 |
| Fish/vegetable fat x 100 | -0.50 | 0.504 | -0.50 | 0.564 | -0.70 | 0.439 |
| Fish/kcal x 100 | -0.10 | 0.942 | -0.80 | 0.416 | -0.60 | 0.523 |
| Fish oil/total fat x 100[a] | -1.30 | 0.141 | -2.50 | 0.016 | -1.80 | 0.092 |
| Fish oil/animal fat x 100[a] | -1.50 | 0.053 | -2.80 | 0.003 | -2.10 | 0.032 |
| Fish oil/animal minus fish fat x 100 | -1.46 | 0.050 | -2.79 | 0.003 | -2.07 | 0.030 |
| Fish oil/vegetable fat x 100 | -0.40 | 0.669 | -1.00 | 0.308 | -0.70 | 0.483 |
| Fish oil/kcal x 100 | -0.40 | 0.661 | -1.50 | 0.165 | -0.80 | 0.463 |

See key to Table I for units. [a]The fish oil is a component of the total and animal fat therefore should be regarded as a proportion of these.

rates and the measures of animal fat, animal minus fish fat, fish and fish oil consumption and ratios of these quantities. The majority of relationships were non-linear and evidence of non-constant variance was also observed in exploratory linear regression analysis. A linear regression analysis was performed using the cancer mortality rate as the dependent variable and the ratio of the fish or fish oil and animal fat consumption as the predictor variable. To ensure the validity of the linearity and constant variance assumptions logarithms to base 2 of the predictor variables were used in the regression analysis; this transformation allowed the regression coefficient to be interpreted as the associated change in the cancer mortality rate for a doubling in consumption of the predictor variables. The validity of the assumptions of constant variance and normally distributed residual were investigated graphically and using the Shapiro Francia W' test respectively.

### Results

Tables II, III and IV show regression coefficients for male CRC, female CRC and female breast cancer respectively for 1983–1987, and the consumption of fat (animal, animal minus fish and vegetable), fish and fish oil, fish and fish oil as a proportion of fat (total, animal minus fish, animal and vegetable) and calories for the time periods 1961–3, 1974–6 and 1984–6. For 1974–6 and 1984–6 the results are essentially similar but those for 1961–3 were somewhat weaker.

As can be seen, all three cancers correlate with animal fat consumption ($P<0.01$) and animal minus fish fat ($P<0.01$) but not with vegetable fat consumption, in all three time periods. Current (1984–86) fish consumption ($P<0.04$) and that 10 years previously (1974–76; $P<0.05$) correlated with male CRC and showed a suggestive correlation with female CRC, though not with female breast cancer. However, when fish and fish oil consumption were analysed as a proportion of total or animal or animal minus fish fat, the correlations became much stronger.

There was no apparent relationship between fish oil and animal minus fish fat consumption. The observed inverse associations betwen the cancer mortality rates and the ratio of fish oil to animal minus fish fat consumption are therefore unlikely to be the result of an inverse relationship to the denominator of the ratio. On average, the animal minus fish fat makes up 98% of the animal fat consumption (range 93–99%) and consequently the regression coefficients are almost identical. As the hypothesis being tested is that a high proportion of fish or fish oil in the diet is protective against the detrimental effects of animal fats the most important correlations are those between cancer mortality and fish oil as a proportion of animal fat intake. Scatterplots for the most important correlations, i.e. for the two cancers against total animal fat and fish and fish oil consumption as a proportion of total animal fat (1984–1986) are shown in Figures 1–3.

Both male and female CRC correlated with both fish and fish oil as a proportion of animal fat consumption and of animal minus fish fat to a similar extent in each of the three time periods (Tables II and III). For fish oil as a proportion of total fat, the regression coefficients were again significant for all three time periods ($P<0.05$), but for fish/total fat the correlations were significant for the current period (1984–86; $P=0.003$ and $P=0.01$; regression coefficients $-2.90$ to $-1.60$) and that 10 years previously but not 23 years previously.

For breast cancer, fish oil as a proportion of animal or animal minus fish fat correlated for all time periods (Table IV), but fish/animal fat was significant only for the 1974–76 period ($P=0.033$; regression coefficient $-1.90$ to $-1.00$). There was no consistent correlation with fish or fish oil as a proportion of total fat and breast cancer. No correlations were found between any of the cancer sites and fish or fish oil/vegetable fat.

The countries were divided arbitrarily into two groups, namely those with an animal fat consumption >85 g caput$^{-1}$ day$^{-1}$ and those with a consumption <85 g caput$^{-1}$ day$^{-1}$. The results for male CRC are shown in Table V. In those countries with a high animal fat intake both fish and fish oil consumption showed an inverse relation with the risk of male CRC, whereas in those countries in which little animal fat is consumed there was no significant effect on this risk. Similar results were observed for female CRC and female breast cancer.

**Figure 1** Relationship between colorectal and breast cancer mortality (1983–87) and total animal fat consumption (1984–86) in 24 European countries. Points labelled as in Table I.

**Table V** Effect of current fish and fish oil consumption on male CRC (1984–86) in countries with a high animal fat intake and a low animal fat intake

| *Level of animal fat intake* | *Fish consumption* Regression coefficient | P | *Fish oil consumption* Regression coefficient | P |
|---|---|---|---|---|
| High: ≥85 g caput$^{-1}$ day$^{-1}$ | −3.33 | <0.001 | −3.53 | 0.001 |
| Low: <85 g caput$^{-1}$ day$^{-1}$ | 1.43 | 0.053 | 1.56 | 0.465 |

**Figure 2** Relationship between colorectal and breast cancer mortality (1983–87) and fish consumption as a proportion of total animal fat consumption (1984–86) in 24 European countries. Points labelled as in Table I.

**Figure 3** Relationship between colorectal and breast cancer mortality (1983–87) and fish oil consumption as a proportion of total animal fat consumption (1984–86) in 24 European countries.

Although this was a European study, the two North American countries of USA and Canada were added for comparison. When data for the current period from these two countries were included in the analyses the regression coefficients for fish/animal fat were −2.90 ($P<0.001$) for male CRC, −1.70 ($P=0.002$) for female CRC and −1.53 ($P=0.061$) for female breast cancer; those for fish oil/animal fat were −3.23 ($P=0.002$) for male CRC, −1.82 ($P=0.007$) for female CRC and −2.19 ($P=0.021$) for female breast cancer. For both male and female CRC the individual points for the two North American countries fell within the confidence limits in Figures 2 and 3 and for female breast cancer they were close to them. Similarly, including data for these two North American countries for the other time periods had virtually no effect on the regression coefficients.

Both the USA and Canada fall in the group with a high animal fat intake of $\geqslant 85$ g caput$^{-1}$ day$^{-1}$. When included in the analysis used for Table V, the regression coefficients in the high animal fat intake countries were −3.34 ($P<0.001$) for fish consumption and −3.21 ($P=0.003$ for fish oil consumption).

### Discussion

In a preliminary report (Caygill and Hill, 1995) we concluded from a correlation study of 24 European countries that fish and fish oil consumption showed an inverse relation with CRC, but not with female breast cancer. The apparent protection was stronger for male than for female CRC, and for current intakes compared with those 23 years previously.

While that paper was in press, Bartram *et al.* (1995) published a paper where they suggested in their discussion that the fish oil consumption *per se* was less important than when expressed as a proportion of total fat. This suggestion was attractive since it could take account of the opposing putative effects of *n*-3 PUFAs ('protective') and animal fat ('tumour-promoting'). If *n*-3 PUFAs actually protected against tumour-promoting effects of animal fat then its effect would only be apparent when the 'challenge' was sufficiently great. We therefore extended our study to include fish and fish oil consumption as a proportion of total fat, animal fat, animal minus fish fat and vegetable fat. In addition to this effect of total fat intake on the ratios, we have also divided the countries into those with a high animal fat ($\geqslant$85 g caput$^{-1}$ day$^{-1}$) and low animal fat ($<$85 g caput$^{-1}$ day$^{-1}$) intake and analysed the two groups separately.

As observed in population studies by many groups previously, CRC incidence was strongly correlated with animal fat intake but not at all with vegetable fat intake. The apparent protective effect of consumption of fish and fish oil *per se* was lower than that of fish or fish oil as a proportion of either animal, animal minus fish or total fat. There was no apparent protective effect of fish or fish oil as a proportion of vegetable fat consumption.

The strongest apparent protective effect was observed for fish oil as a proportion of animal or animal minus fish fat intake: this was seen for both male and female CRC, and in all three time periods of consumption. The apparent protective effect was only marginally less strong for fish consumption as a proportion of animal minus fish fat. The apparent protective effect of fish oil as a proportion of total fat was weaker than that for fish oil/animal minus fish fat. In fact, the effect of fish oil/total fat (and also of fish oil/total energy intake) is probably secondary to the effect of fish oil/animal minus fish fat, since there was no apparent protective effect of fish oil/vegetable fat.

This is an ecological study and so has a range of potential confounding factors. Whenever populations are compared they differ not only in their intake of dietary fat but also in racial factors, climate, cultural factors and a range of dietary factors. By comparing only European countries, the effect of variations in racial and cultural factors on colorectal and breast cancer risk should be minimised (in comparison, for example, with a study that included African and Asian as well as European countries). There is a wide range of climate within Europe, from Arctic to Mediterranean, from coastal to mid-continental types though without tropical and monsoon type climates. Climatic factors may be important in determining energy requirements and in the balance between energy intake and energy utilisation. Fat intake is, of course, related to total energy intake, which itself is a potential confounder. A major confounding factor in comparisons of populations is the variation in the quality of the data used. By limiting the study to European countries and by using data from a common source, these variations are reduced to a minimum. However, addition of the United States and Canada made no difference to the analysis, and the data were entirely consistent with the European data.

As all of these effects are only apparently seen in countries with a high fat intake, on the basis of our results, dietary recommendations on fish consumption in countries with low animal fat intakes are irrelevant with respect to colorectal and breast cancer prevention.

If fish oil is postulated as protecting in some way against the detrimental effects of animal fat, then it is plausible to consider that protection is only relevant to countries with a high animal fat intake. From the regressions in Tables III–V we can estimate the postulated effects of changes in fish or fish oil intake combined with decreased animal fat intake. In *Health of the Nation* (Department of Health, 1992) the dietary target, in the interests of coronary heart disease prevention, is a 15% decrease in animal fat intake. This might be expected (see Table II) to yield a 6% decrease in male CRC mortality. This, combined with a 3-fold increase in fish oil intake might possibly decrease male CRC mortality by as much as 30% if the hypothesis is correct. A 3-fold increase in fish oil intake could be achieved, either by increased fish consumption to approximately three times per week or by the consumption of two standard fish oil capsules per day (which may be more easily achieved in view of falling fish stocks).

The potential implications of these observations highlight the need for their confirmation by analytical epidemiological studies (such as that of Willett *et al.*, 1990).

### References

ANTI M, MARRA G AND AMALEO F. (1992). Effect of n-3 fatty acids on rectal mucosal proliferation in subjects at risk for colon cancer. *Gastroenterology*, **103**, 883–891.

BARTRAM H-P, GOSTNER A, REDDY BS, RAO CV, SCHEPPACHN W, DUSL G, RICHTER A, RICHTER F AND KASPER H (1995). Missing anti-proliferative effect of fish oil on rectal epithelium in healthy volunteers consuming a high fat diet; potential role of the n3:n6 fatty acid ratio. *Eur. J. Cancer Prev.*, **4**, 231–238.

BOUTRON MC, WILPART M AND FAIVRE J. (1991). Diet and colorectal cancer. *Eur. J. Cancer Prev.*, **1**, (Suppl.2),13–20.

CARROLL KK. (1983). The role of dietary fat in carcinogenesis. In *Dietary Fats and Health. Am. Oil Chem. Soc.*, Perkins EG, Viseck WJ. (eds), pp. 710–720. Champain, IL.

CAYGILL CPJ AND HILL MJ. (1995). Fish n-3 fatty acids and human colorectal and breast cancer. *Eur. J. Cancer Prev.*, **4**, 329–332.

CLEVELAND WS. (1979). Robust locally weighted regression and smoothing scatterplots. *J. Am. Stat. Assoc.*, **74**, 829–836.

DEPARTMENT OF HEALTH. (1992). *The Health of the Nation: a Strategy for Health in England.* HMSO: London.

DRASAR BS AND IRVING D. (1973). Environmental factors and cancer of the colon and breast. *Br. J. Cancer*, **27**, 167–172.

FAO (FOOD AND AGRICULTURE ORGANIZATION OF THE UNITED NATIONS). (1991). *Food Balance Sheets, 1984–86 Average.* FAO: Rome.

GONZALEZ MJ, SCHEMMEL RA, DUGAN L JR, GRAY JI AND WELSCH CW. (1993). Dietary fish oil inhibits human breast carcinoma growth: a function of increased lipid peroxidase. *Lipids*, **28**, 827–832.

HILL MJ. (1987). Dietary fat and human cancer. *Anticancer Res.*, **7**, 281–293.

HURSTING SD, THORNQUIST M AND HENDERSON MM. (1990). Types of dietary fat and the incidence of cancer at five sites. *Prev. Medm.*, **19**, 242–253.

KAIZER L, BOYD NF, KRIUKOV V AND TRITCHLER D. (1989). Fish consumption and breast cancer risk: an ecological study. *Nutr. Cancer*, **12**, 61–68.

LEVI F, LA VECCHIA C, LUCCHINI F AND BOYLE P. (1993). Cancer incidence and mortality in Europe, 1983–87. *Med. Soc. Prev.*, **38**, (supp.3).

LEVI F, LUCCHINI F AND LA VECCHIA C. (1994). Worldwide patterns of cancer mortality. *Eur. J. Cancer Prev.*, **3**, 109–143.

LINDNER MA. (1991). A fish oil diet inhibits colon cancer in mice. *Nutr. Cancer*, **15**, 1–11.

REDDY BS, COHEN LA, MCCOY GD, HILL P, WEISBURGER JH AND WYNDER EL. (1980). Nutrition and its relationship to cancer. *Adv. Cancer Res.*, **32**, 237–245.

REDDY BS, BURRILL C AND RIGOTTY J. (1991). Effect of a diet high in omega-3 and omega-6 fatty acids on initiation and postinitiation stages of colon carcinogenesis. *Cancer Res.*, **51**, 487–491.

SASAKI S, HORACSEK M AND KESTELOOT H. (1993). An ecological study of the relationship between dietary fat intake and breast cancer mortality. *Prev. Med.*, **22**, 187–202.

VISEK WJ AND CLINTON SK. (1983). Dietary fat and breast cancer. In *Dietary Fats and Health. Am Oil Chem. Soc.*, Perkins EG, Visek WJ. (eds). pp. 721–740. Champain, IL.

WILLETT WC, STAMPFER MJ, COLDITZ GA, ROSNER BA AND SPEIZER FE. (1990). Relation of meat, fat and fiber intake to the risk of colon cancer in a prospective study among women. *N. Engl. J. Med.*, **323**, 1664–1672.